# Salivary MicroRNA in Pancreatic Cancer Patients

**DOI:** 10.1371/journal.pone.0130996

**Published:** 2015-06-29

**Authors:** Marine Humeau, Alix Vignolle-Vidoni, Flavie Sicard, Frédéric Martins, Barbara Bournet, Louis Buscail, Jérôme Torrisani, Pierre Cordelier

**Affiliations:** 1 Inserm, UMR1037 CRCT, F-31000 Toulouse, France; 2 Université Toulouse III-Paul Sabatier, UMR1037 CRCT, F-31000 Toulouse, France; 3 Department of Gastroenterology, CHU Toulouse- Rangueil, Toulouse, France; 4 Department of Surgery, CHU Toulouse- Rangueil, Toulouse, France; 5 INSERM U1048, F-31000 Toulouse, France; National University of Singapore, SINGAPORE

## Abstract

**Background:**

Pancreatic cancer is the fourth leading cause of cancer death in Western countries, with the lowest 1-year survival rate among commonly diagnosed cancers. Reliable biomarkers for pancreatic cancer diagnosis are lacking and are urgently needed to allow for curative surgery. As microRNA (miRNA) recently emerged as candidate biomarkers for this disease, we explored in the present pilot study the differences in salivary microRNA profiles between patients with pancreatic tumors that are not eligible for surgery, precancerous lesions, inflammatory disease or cancer-free patients as a potential early diagnostic tool.

**Methods:**

Whole saliva samples from patients with pancreatic cancer (n = 7), pancreatitis (n = 4), IPMN (n = 2), or healthy controls (n = 4) were obtained during endoscopic examination. After total RNA isolation, expression of 94 candidate miRNAs was screened by q(RT)PCR using Biomark Fluidgm. Human-derived pancreatic cancer cells were xenografted in athymic mice as an experimental model of pancreatic cancer.

**Results:**

We identified hsa-miR-21, hsa-miR-23a, hsa-miR-23b and miR-29c as being significantly upregulated in saliva of pancreatic cancer patients compared to control, showing sensitivities of 71.4%, 85.7%, 85,7% and 57%, respectively and excellent specificity (100%). Interestingly, hsa-miR-23a and hsa-miR23b are overexpressed in the saliva of patients with pancreatic cancer precursor lesions. We found that hsa-miR-210 and let-7c are overexpressed in the saliva of patients with pancreatitis as compared to the control group, with sensitivity of 100% and 75%, and specificity of 100% and 80%, respectively. Last hsa-miR-216 was upregulated in cancer patients as compared to patients diagnosed with pancreatitis, with sensitivity of 50% and specificity of 100%. In experimental models of PDAC, salivary microRNA detection precedes systemic detection of cancer cells markers.

**Conclusions:**

Our novel findings indicate that salivary miRNA are discriminatory in pancreatic cancer patients that are not eligible for surgery. In addition, we demonstrate in experimental models that salivary miRNA detection precedes systemic detection of cancer cells markers. This study stems for the use of salivary miRNA as biomarker for the early diagnosis of patients with unresectable pancreatic cancer.

## Introduction

Pancreatic ductal adenocarcinoma (pancreatic cancer, PDAC) is the fourth leading cause of cancer death in Western countries, with the lowest five-years relative [1] and 1-year survival [2] rates among commonly diagnosed cancers. pancreatic cancer is anticipated to move to the second leading cause of cancer death worldwide by 2020 in the absence of improvements in treatment [3]. There are currently no means for the reliable diagnosis of early stages of pancreatic cancer. Consequently, the vast majority of patients (85%) display an advanced disease that results in a low resection rate (15% of patients) leading to a dismal overall median survival of 4 to 6 months. Thus, discovering biomarkers for early pancreatic cancer diagnosis may favor early patients’ management and prognosis.

MicroRNAs (miRNAs) have recently emerged as a new class of robust biomarkers for cancer diagnosis, including PDAC [4]. These potent regulators of gene expression can be thoroughly quantified in diverse tissues and fluids, due to their inherent high stability as compared to proteins and messenger RNAs. Of importance, miRNAs can be quantified in very low amounts of material, including micro-biopsies, and in highly degraded samples. Recent reports extensively demonstrated that miRNA profiles can successfully discriminate normal from cancerous pancreatic tissue, and may also predict cancer prognosis or response to treatment [4]. The stability of miRNAs has been once again underscored as miRNA profiling in plasma was recently demonstrated to differentiate PDAC patients from healthy controls [4]. Such findings pave the way for the use of circulating miRNAs as minimally-invasive PDAC biomarkers.

Several other body fluids such as urine, semen and saliva have been recently considered as repositories for cancer diagnosis [5,6]. Saliva has the superior advantage as sample collection is simple, non-invasive, causes little anxiety on the part of patients and can be repeated. Saliva has been demonstrated to contain proteins/peptides, nucleic acids, electrolytes, and hormones that originate from both local and systemic sources and recent studies have prompted interest in using saliva as a source of biomarkers. Accordingly, the use of saliva for detection of oral diseases has been extensively demonstrated [7], and saliva recently emerged as a wealthy source of miRNAs, such as has-miR-31, for oral cancer diagnosis [8–11]. On the other hand, saliva use for systemic disease is largely unclear. In recent years, metabolic [12], transcriptomic [13] and microbiota [14] salivary profiles were demonstrated to possess discriminatory power for the detection of PDAC, with high specificity and sensitivity.

To our knowledge, the use of salivary miRNAs for the diagnosis of non resectable pancreatic cancer has not been reported to date. Consequently, the goal of this study was to explore the scientific evidence and provide a rationale for the use of saliva for unresectable PDAC detection that represents the vast majority of patients diagnosed with this cancer. In this pilot study, we found that four salivary miRNAs (hsa-miR-21, hsa-miR-23a, hsa-miR-23b and hsa-miR-29c) successfully segregated PDAC patients from cancer-free donors, while hsa-miR-210 and let-7c indicate pancreatitis and hsa-miR-216 discriminates pancreatitis from cancer. In addition, we demonstrate herein in experimental models of PDAC that salivary miRNA detection precedes detection of systemic cancer cells markers. Taken together, we present preliminary data that shows significant differences in miRNA profiles between saliva from patients with PDAC and saliva from patients that are tumor-free. The discovered salivary biomarkers possess inherent discriminatory potential for a noninvasive diagnostic tool for PDAC, in patients that are not eligible for surgery.

## Materials and Methods

### Patients

This protocol was approved by the Ethical Committee (*Comité de Protection des Personnes Sud-Ouest et Outre Mer N°1*, *number 1-10-21*). To avoid blood contamination, patients were asked not to brush their teeth within 45 minutes prior to sample collection. Saliva was collected using sterile tips and micropipettes during endoscopic examination under general anesthesia with propofol. Saliva was immediately placed in pre-chilled 1.5-ml microcentrifuge tubes containing and equal volume of Saliva protect reagent (Qiagen) and stored at -80°C until ready for use. In this pilot study, we included patients aged >18 years who had given their written informed consent. Other criteria for inclusion were no contraindications for general anesthesia or for endoscopic ultrasound. Fine needle aspiration material was used for histological, cytological and molecular (KRAS activating mutation analysis[15]) diagnosis of pancreatitis or pancreatic cancer. Twenty-one patients were included in this study; 7 were diagnosed with locally advanced, unresectable pancreatic cancer, 4 were diagnosed with pancreatitis (either acute or chronic) and 4 had unrelated digestive diseases (control group) (Table 1). Patients diagnosed with intraductal papillary mucinous neoplasia (IPMN) (n = 2) were also included. Patients were not treated before saliva collection.

**Table 1:** Patients’ characteristics.

**Group:**	Control			
	**Patient #**	**Age**	**Diagnostic**	
	13	64	colon polyps	
	14	81	Gallstones	
	15	70	colon polyps	
	16	66	Irritable bowel syndrome	
	mean	70		
		(64–81)		
**Group:**	Pancreatitis			
	**Patient #**	**Age**	**Diagnostic**	
	4	54	Chronic pancreatitis	
	17	39	Acute pancreatits	
	18	51	Acute pancreatits	
	19	54	Chronic pancreatitis	
	mean	50		
		(39–54)		
**Group:**	Cancer			
	**Patient #**	**Age**	**Diagnostic**	**KRAS status**
	3	59	Pancreatic adenocarcinoma	positive
	6	66	Pancreatic adenocarcinoma	positive
	7	66	Pancreatic adenocarcinoma	negative
	8	68	Pancreatic adenocarcinoma	positive
	9	68	Pancreatic adenocarcinoma	positive
	10	67	Pancreatic adenocarcinoma	positive
	12	74	Pancreatic adenocarcinoma	positive
	mean	67		
		(59–74)		
**Group:**	Benign pancreatic masses			
	**Patient #**	**Age**	**Diagnostic**	
	21	52	IPMN (secondary branch ducts)	
	22	83	IPMN (mixed)	

### Experimental protocol

All animals experiments were conducted according to the national ethical guidelines for experimental research and protocol were approved by the regional ethical committee of Anexplo UMS 006 for animal experimentation and were performed in accordance with the Guide for the Care and Use of Laboratory Animals (US National Institutes of Health). Human pancreatic cancer-derived Mia PACA-2 cells expressing secreted Lucia luciferase [16,17] are grown in RPMI medium supplemented with 10% fetal calf serum, L-glutamine, an antibiotic, an antimycotic cocktail (Life Technologies), and Plasmocin (InvivoGen) in a humidified incubator at 37°C in 5% CO_2_. Six two-week-old female nu/nu mice were anesthetized by intraperitoneal injection of pentobarbital (80mg/kg) diluted in 0.9% NaCl, supplemented with oral anaesthesia using oxygen/isofluorane (2.5 mixture) and Mia PACA-2 Lucia cells were implanted in the tail of pancreas as previously described [16,17]. Saliva secretion was not stimulated by pilocarpine. Saliva was obtained from the oral cavity by micropipette and immediately placed in pre-chilled 1.5-ml microcentrifuge tubes containing and equal volume of Saliva protect reagent (Qiagen). Collection was completed in 20 minutes and samples were stored at −80°C until analyzed. For non-invasive tracking of tumor growth, blood was sampled by retro-orbital collection and centrifuged at 1000 ×*g* for 10 min in microcentrifuge tubes treated with EDTA. Lucia production was measured in 5μl of plasma using coelenterazine (50μM) as a substrate. For miRNA quantification studies, tumors were frozen in liquid nitrogen and stored at -80°C until use. At the end of the experiments, mice were killed by injection of a lethal dose of pentobarbital.

### RNA extraction

Before saliva samples were used, they were defrosted on ice and centrifuged for 15 minutes at 2600 x*g* at 4°C. The cell free supernatant was collected from the pellet and used immediately in the next step. Total RNA was isolated from 250 μL saliva supernatant and from tumors using Trizol LS reagent (Life technologies) and miRNAeasy extraction kit (Qiagen), respectively. DNase I treatment (DNase I, Qiagen) was used to remove contaminating DNA during RNA extraction. The concentration of total RNA was measured using Nanodrop N-100.

### miRNA quantification

Total salivary, cellular or tumor RNA (20ng) was reverse transcribed and pre-amplified using the Universal cDNA synthesis kit (Exiqon), followed by Specific Target Amplification (STA) using TaqMan PreAmp Master Mix (Life technologies) and pooled 94 microRNA LNA PCR primer sets (Exiqon, listed in S1 Table). Following 15 pre-amplifaction cycles, STA reactions were diluted 1:10 in nuclease free water. qPCR Assay Mix consisted of TaqMan Gene Expression Master Mix (Life technologies), DNA Binding Dye Sample Loading Reagent (Fluidigm), EvaGreen (Biorad), Forward and Reverse primer mix (Exiqon) and Assay Loading Reagent, and prepared as per the manufacturer’s recommendations. Samples and sample mix was loaded on a Fluidigm chip (Fluidgm) and quantitative real time PCR reaction was run at 95°C for 10 minutes, followed by 30 cycles at 95°C for 10 seconds and 60°C for 1 minute on the Fluidigm platform (Fluidgm). The quantification cycle (Cq) value is defined as the cycle number in the fluorescence emission, which exceeds that of a fixed threshold. A Cq of 15 to 30 was considered high expression and a Cq of 35 is considered low expression. A Cq value more than 40 was considered as undetectable miRNA. Data normalization was conducted using RQ manager 1.2.1 and Data Assist v3.0 from Applied Biosystems.

### Statistical analysis

The qPCR-based gene expression values between the different groups were compared using the nonparametric Wilcoxon rank-sum test. Candidate biomarker miRNAs were then selected based on P < 0.05.

## Results

### Identification of pancreatic cancer-specific salivary miRNAs

For this pilot study, 94 miRNAs were selected from the literature as follow: previously reported biomarkers for cancer, previously reported biomarkers for pancreatic cancer, detected in blood of patients with cancer or detected in saliva of patients with cancer (S1 Table). Expression of candidate miRNAs was screened by q(RT)PCR using Biomark Fluidgm in patients with pancreatic cancer (n = 7), pancreatitis (n = 4), intraductal papillary mucinous neoplasia (IPMN, n = 2) or without cancer (n = 4) (Table 1).

**Fig 1:**
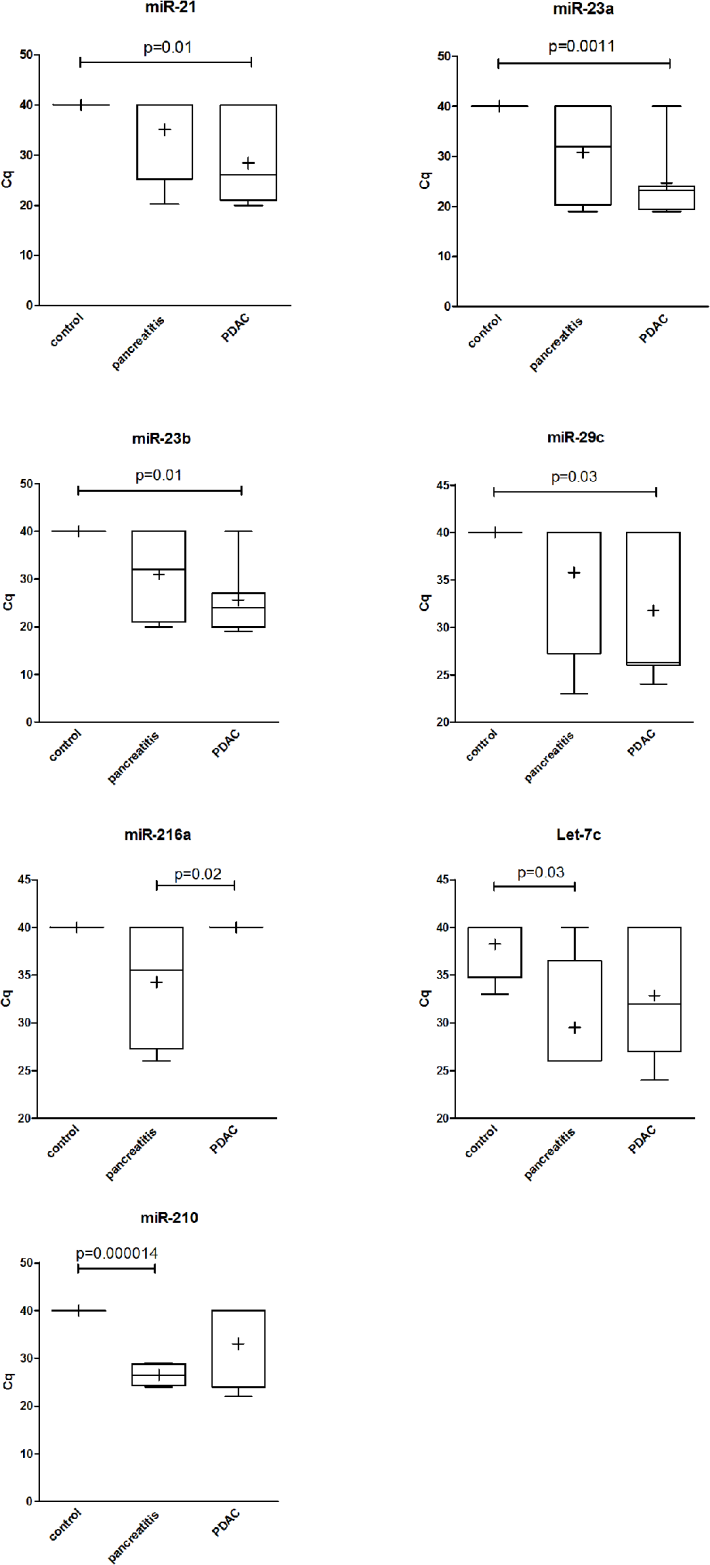
Analysis of candidate miRNAs expression (Cq) in the saliva of patients with unresectable pancreatic cancer (n = 7), pancreatitis (n = 4) or cancer-free patients (n = 4). Results are presented as Whiskers box (min-max) and mean (+) is indicated. The *p* value (nonparametric Wilcoxon rank-sum test) is indicated.

Of the 94 miRNAs, 23 miRNAs were undetectable in all samples tested (S2 Table). We found that 4 miRNAs (hsa-miR-21, hsa-miR23a, hsa-miR-23b and hsa-miR-29c) were significantly expressed in saliva from patients with pancreatic cancer (n = 7), while undetectable in the saliva of control patients (n = 4; Wilcoxon test, 0.001< *p* < 0.03) (Fig 1 and Table 2). The expression of the candidate miRNAs was strictly specific of pancreatic cancer (100%) with excellent sensitivity (ranging from 57% to 86%, Table 2). The candidate miRNAs were also detected within saliva of patient diagnosed with other cancers (n = 2, Table 2), while hsa-miR23a and hsa-miR-23b were detected in the saliva of patients diagnosed with IPMN, a well-characterized precursor lesion of PDAC. Of note, hsa-miR-21, hsa-miR23a, hsa-miR-23b and hsa-miR-29c could be detected in the saliva of patients with pancreatitis (Fig 1).

**Table 2:** Average Cq values, sensitivity and specificity of the candidate microRNAs.

	Cancer		Control				
	mean Cq	SD	mean Cq	SD	*p*	spécificity	sensitivity
hsa-miR-21	28,00	3,10	40,00	0,00	0,012	100%	71%
hsa-miR-23a	24,90	2,63	40,00	0,00	0,001	100%	86%
hsa-miR-23b	25,97	2,55	36,75	3,25	0,014	100%	86%
hsa-miR-29c	31,76	2,92	40,00	0,00	0,03	100%	57%

The expression of miRNAs in whole saliva from patients with PDAC (n = 7) were compared to the expression of miRNAs in whole saliva from patients without cancer (n = 4). The *p* value (nonparametric Wilcoxon rank-sum test) is indicated.

Pancreatitis is a common inflammation of the pancreas. Despite modern imaging techniques, difficulties persist to differentiate PDAC from benign diseases such as chronic pancreatitis especially in its pseudotumoral form [15]. Such consideration is critical to avoid unnecessary resection of benign lesions (such as focal lesions of chronic pancreatitis or autoimmune pancreatitis) or to delay the treatment of PDAC in a subset of patients. We previously demonstrated that RNA signatures [18] or *KRAS* mutation analysis [15,19] may be helpful for diagnostic. In the present work, we explored whether salivary miRNA may represent a non-invasive screening method for pancreatitis detection. We found that salivary hsa-miR-216 may help discriminate pancreatitis from PDAC, with excellent specificity (100%), but poor sensitivity (50%) (Table 3). On the other hand, hsa-miR-210 and let-7c are overexpressed in the saliva of patients diagnosed with pancreatitis, but could not be detected in the saliva of control patients (Table 4). In addition, hsa-miR-210 presents remarkable specificity and sensitivity for pancreatitis, either chronic or acute (100%, Table 3). On the other hand, hsa-miR-210 was detected in the saliva of patients with PDAC. Taken together, our pilot study strongly suggests that salivary miRNAs could be useful for the diagnosis of pancreatitis and non resectable PDAC.

**Table 3:** Average Cq values, sensitivity and specificity of the candidate microRNA.

	Cancer		Pancreatitis				
	mean Cq	SD	mean Cq	SD	*p*	spécificity	sensitivity
hsa-miR-216	40,00	0,00	34,25	3,47	0,024	100%	50%

The expression of hsa-miR-216 in whole saliva from patients with PDAC (n = 7) were compared to the expression of miRNAs in whole saliva from patients with pancreatitis (n = 4). The *p* value (nonparametric Wilcoxon rank-sum test) is indicated.

**Table 4:** Average Cq values, sensitivity and specificity of the candidate microRNAs.

	Conrol		Pancreatitis				
	mean Cq	SD	mean Cq	SD	*p*	spécificity	sensitivity
hsa-miR-210	40,00	0,00	26,50	1,19	0,000014	100%	100%
Let-7c	38,25	1,75	29,50	3,50	0,033	75%	80%

The expression of hsa-miR-216 in whole saliva from patients with pancreatitis (n = 4) were compared to the expression of miRNAs in whole saliva from control patients (n = 4). The *p* value (nonparametric Wilcoxon rank-sum test) is indicated.

### Salivary miRNAs precede protein-based, systemic detection of PDAC in experimental models

**Fig 2:**
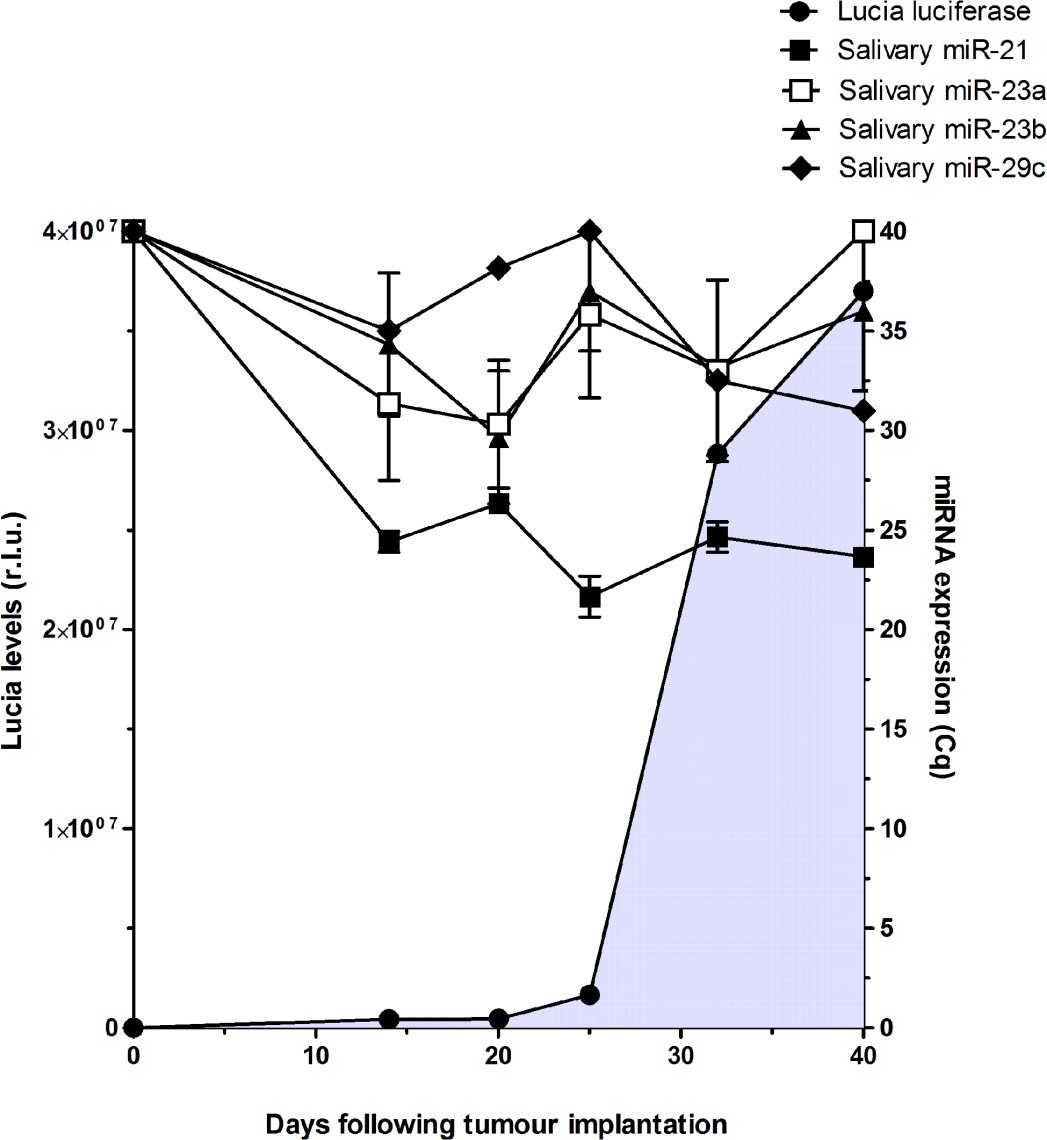
Analysis of salivary hsa-miR-21, hsa-miR-23a, hsa-miR-23b and hsa-miR-29c levels and Lucia blood levels in mice xenografted with Mia PACA-2 Lucia cells at the time indicated following tumor induction. Results are mean ± S.D. of 6 biological replicates done in experimental triplicates. miRNA levels are expressed in Cq, Lucia levels are expressed in relative light units (r.l.u.). The grey zone corresponds to tumor detection using secreted Lucia as a systemic, protein-based tumor marker.

We next investigated the kinetic of salivary miRNA detection in experimental model of pancreatic cancer. Mia PACA-2 human-derived pancreatic cancer cells were implanted in the pancreas of athymic mice (n = 6). We found that these cells and resulting xenografts express high levels of hsa-miR-21, hsa-miR-23a, hsa-miR-23b and hsa-miR-29c (S3 and S4 Tables). These cells were engineered to express high-levels of secreted luciferase for protein, systemic-based, non-invasive tumor monitoring [16,17]. Experimental pancreatic cancer tumors were detected 25 days following tumor cell engraftment using systemic dosage of secreted luciferase and before they became palpable (Fig 2).

Interestingly, hsa-miR-21 was readily detected at high levels in saliva from tumor-bearing mice, as soon as 14 days following tumor induction (mean Cq = 24.41 ±1.29 Fig 2 and S5 Table), while undetectable in the saliva of tumor-free animals (data not shown). In addition, salivary hsa-miR-21 expression remained elevated during the course of the experiment (Fig 2). On the other hand, salivary hsa-miR-23a, hsa-miR-23b and hsa-miR-29c were detected at low levels in the saliva of PDAC-bearing mice (Fig 2 and S5 Table). Thus, we validate hsa-miR-21 as a salivary biomarker in this experimental model of PDAC; in addition our results strongly suggest that salivary miRNA are more sensitive than systemic protein markers for the diagnosis of pancreatic tumors.

## Discussion

A major issue in pancreatic cancer research is the need of biomarkers for early diagnosis, not only for the early detection of the disease in cohort of patients, but also to accelerate decision making in difficult-to-diagnose pancreatic masses. This is extremely important considering that patients’ survival and prognosis depend on the stage of the tumor at the time of diagnosis. Theoretically, early diagnosis can allow for tumor resection and is usually associated with the best prognosis. However, the difficulty of early diagnosis and the high prevalence of metastasis associated with pancreatic cancer contribute to its dismal prognosis [1]. Thus, the past few years have witnessed intensive study in searching for more sensitive, specific and cost-effective biomarkers. To date, many molecular-based, multi-omics strategies are utilized to achieve this goal. Tissue miRNAs were recently demonstrated as novel biomarkers for the diagnosis, prognosis and prediction to treatment response for pancreatic cancer patients [4]. Remarkably, these small noncoding RNAs can also be detected in many if not all body fluids [20]. Accordingly, miRNA profiling in blood was recently demonstrated to differentiate cancer patients from healthy controls [4], and circulating miRNA analysis have been increasingly suggested as a novel biomarker for pancreatic cancer diagnosis.

In the past few years, miRNAs in human saliva have been demonstrated to be potential biomarkers for diagnosis purposes. Because collection is non-invasive, atraumatic and easily accessible, using saliva for early disease detection is ideal. Historically, hsa-miR-31 was one of the first discriminatory miRNA salivary biomarkers identified for oral cancer [21]. Recently, over-expression of has-miR-17 and has-miR-20a have been reported to be significantly associated with poor outcome of salivary adenoid cystic carcinoma [22]. In addition, 13 miRNAs were found significantly deregulated in saliva of oral squamous cell carcinoma patients as compared to healthy controls [23]. Last, salivary miRNA profiles differ in saliva from patients with malignant from saliva from patients with a benign parotid gland tumor, and thus represent a new non-invasive diagnostic tool for diagnosing tumors in the salivary glands [9]. During the redaction of this manuscript, Xie *et al* described that salivary miR-3679-5p and miR-940, two newly characterized miRNAs that were not studied in the present work, may be specific of patients with resectable PDAC, with reasonable specificity and sensitivity [24]. On the other hand, saliva use for miRNA detection has not been evaluated to date in unresectable PDAC patients that represent the vast majority (85%) of patients diagnosed with this cancer.

In the present proof-of-concept study, we collected saliva from patients with unresectable pancreatic cancer (n = 7), pancreatitis (n = 4), IPMN (n = 2), and cancer-free patients (n = 4) undergoing endoscopic examination. Of more than 90 miRNAs tested, 4 were identified as being significantly deregulated in saliva of pancreatic cancer patients compared to control (hsa-miR-21, hsa-miR-23a, hsa-miR-23b and hsa-miR-29c). In addition, hsa-miR-21, hsa-miR-23a and hsa-miR-23b were strictly specific to cancer patients, with excellent sensitivity (71.4% and 85.7%, respectively). On the other hand, Let-7c and hsa-miR-210 were absent in the saliva of control patients but readily detectable in the saliva of patients with pancreatitis, with exquisite specificity and selectivity (hsa-miR-210).

However, at this stage of this project, salivary testing failed to differentiate between pancreatitis and PDAC, as hsa-miR-216 is detected only in pancreatitis and not in cancer, but with poor sensitivity. Taken together, we demonstrate for the first time that salivary miRNA are indicative of pancreatic disease and can be used to diagnose unresectable PDAC (hsa-miR-21, hsa-miR-23a, hsa-miR-23b) or pancreatitis (hsa-miR-210). Hsa-miR-21, hsa-miR-23a and hsa-miR-23b were found significantly deregulated in the saliva of resectable PDAC patients as compared to healthy control during the discovery phase, but were not further investigated as they didn’t exhibit at least a 4-fold change in expression between the two groups [24].

In this work, we have started exploring whether salivary miRNAs may help for the diagnosis of population at risk of developing pancreatic cancer, and thus could be used as marker to prevent tumor incidence. Intraductal papillary mucinous neoplasms (IPMNs) are non-invasive precursor lesions of pancreatic cancer. Recently, miRNAs in cyst fluid have been demonstrated to identify high grade IPMN that requires resection and to exclude non-mucinous cysts implying conservative management with high sensitivity and specificity [25]. We have obtained preliminary results suggesting that hsa-miR-23a and hsa-miR-23b are also be present in saliva from patients diagnosed with IPMN, and could be used for decision making in IPMN management.

However, our study tends to indicate that hsa-miR-21, hsa-miR-23a and hsa-miR-23b are present in the saliva of patients with pancreatitis, while hsa-miR-210 is detected in the saliva of a fraction of patients with PDAC. In addition, hsa-miR-23a and hsa-miR-23b are present in the saliva of patients with IPMN. This could be easily explained as pancreatitis and IPMN are two-well characterized PDAC precursor lesions, indicating that PDAC positive for hsa-miR-210, or hsa-miR-23a and hsa-miR-23b, may have derived from pancreatitis or IPMN, respectively. On the contrary, patients diagnosed with pancreatitis and elevated salivary hsa-miR-21, hsa-miR-23a and hsa-miR-23b, or patients diagnosed with IPMN and elevated salivary hsa-miR-23a and hsa-miR-23b may be at-risk of developing PDAC and may require careful clinical follow-up. We are aware that the present study suffers from small sample sizing and requires an external validation population. Consequently, we have recently constituted the first clinically annotated cohort of pancreatic cancer patients’ samples from different institutes (the BACAP initiative, http://www.chu-toulouse.fr/-projet-bacap-). Such cohort will be immensely informative for further validation and future clinical application of our method, because it represents a unique source of PDAC samples, but also because it’s recapitulate the “natural history” of this disease. Such cohort may help to establish salivary miRNAs, together with additional clinical variables, as novel biomarkers for pancreatic cancer patients’ management. In addition, we have yet to perform comparative studies between different cancer patients to justify that the biomarkers we identified herein are specific for pancreatic cancer.

In this article, we have identified hsa-miR-21, hsa-miR-23a and hsa-miR-23b that were differently expressed between saliva samples of patients with a malignant tumor and cancer-free patients, with excellent specificity and sensitivity. While hsa-miR-21 is also associated with many physiological conditions including but not restricted to cardiovascular and pulmonary diseases, including cardiac and pulmonary fibrosis as well as myocardial infarction, but also with immunological and developmental processes [26], hsa-miR-21 is one of the most cited miRNA in oncology [27], including pancreatic cancer [4]. We previously demonstrated that hsa-miR-21 is early expressed during pancreatic carcinogenesis [28], and that targeting hsa-miR-21 provokes tumor regression in experimental models of pancreatic cancer [17]. Strikingly, hsa-miR-21 appears to be constantly up regulated in pancreatic cancer, and to be indicative of poor survival, response to treatment and/or metastatic disease [4]. In addition, a recent meta analysis recently demonstrated circulating hsa-miR-21 prognostic rather than diagnostic value in different cancers [29]. In the present study, we speculate that salivary hsa-miR-21 may also be of interest for pancreatic cancer diagnosis, and complete the previous characterization of salivary hsa-miR-21 for the detection of esophageal cancer [10]. To our knowledge, we provide herein the first demonstration that hsa-miR-23a and hsa-miR-23b could be detected in the saliva of patients diagnosed with cancer; however, the specificity of both candidate miRNAs for PDAC is still to be demonstrated. Hsa-miR-23a has recently been associated with KRAS [30] and C-MYC [31] mediated signaling pathway, and described as a candidate driving miRNA in pancreatic cancer [30]. Hsa-miR-23a has also been linked to impaired NK cell cytotoxicity [32], EMT [33] and resistance to treatment [34–36]. Interestingly, hsa-miR-23b was recently demonstrated to regulate autophagy associated with radioresistance of pancreatic cancer cells [37].

We next investigated the kinetic of detection of the salivary miRNAs in an experimental model of pancreatic cancer. While hsa-miR-23a and hsa-miR-23b were highly expressed in human pancreatic cancer cells-derived xenografts, they were barely detectable in saliva in this model of tumor-bearing mice. On the other hand, hsa-miR-21 was readily detected in tumors and in saliva of mice xenografted with human pancreatic cancer-derived cells, while undetectable in control animals. This latter finding strongly suggest that salivary hsa-miR-21 originates from experimental tumors, probably *via* tumor-derived exosomes, as recently described [38]. In addition, we demonstrate herein that salivary hsa-miR-21 detection precedes detection of cancer-cell specific tumor marker in this experimental model of PDAC. This strongly suggests that salivary miRNA, including hsa-miR-21, are more sensitive than systemic-based protein markers for the diagnosis of PDAC.

## Conclusion

Taken together, we demonstrate herein for the first time that salivary miRNA could be valuable biomarkers for distinguishing patients with unresectable PDAC from healthy controls, and that salivary miR-210 may help detect pancreatitis. While multicenter studies with larger sample sizes are needed, this work stems for the use of salivary miRNA as novel biomarkers for the diagnosis of unresectable PDAC.

## Supporting Information

S1 Table miRNA quantified in this study. (XLSX)

S2 Table miRNAs Cq values in whole saliva from patients without cancer (n = 4), begning pancreatitis (n = 4), pancreatic adenocarcinoma (n = 7) or IPMN (n = 2). (XLSX)

S3 Table Candidate miRNAs Cq values from Mia PACA-2 Lucia cells (n = 3). (XLSX)

S4 Table Candidate miRNAs Cq values from n = 6 experimental pancreatic tumours (ET). (XLSX)

S5 Table Secreted Luciferase and salivary candidate miRNAs Cq values from n = 6 mice with experimental pancreatic tumours. (XLSX)
